# Macular Oedema Related to Idiopathic Macular Telangiectasia Type 1 Treated with Dexamethasone Intravitreal Implant (Ozurdex)

**DOI:** 10.1155/2014/231913

**Published:** 2014-06-17

**Authors:** Mohamed Loutfi, Thomas Papathomas, Ahmed Kamal

**Affiliations:** ^1^University of Liverpool Medical School, Liverpool L69 3BX, UK; ^2^Eye Unit, Royal Cornwall Hospital, Treliske, Truro TR1 3LJ, UK; ^3^Ophthalmology Department, Aintree University Hospital, Longmoor Lane, Liverpool L9 7AL, UK

## Abstract

A 65-year-old female presented with visual disturbance in her right eye lasting for over 2 months. Following investigations, she was diagnosed with MacTel type 1 in the right eye. Visual symptoms were refractory to initial treatment with intravitreal bevacizumab and thereafter intravtireal triamicinolone. The patient was then treated with Ozurdex, following which central macular thickness (CMT) decreased (from 397 *μ*m to 286 *μ*m) and visual acuity deteriorated (from logMAR 0.48 to 0.59). At 14 weeks posttreatment with Ozurdex, a recurrence of cystoid macular oedema (CMO) was observed. Following a second Ozurdex, visual acuity improved (from logMAR 0.7 to 0.64) and CMT decreased (from 349 *μ*m to 279 *μ*m). An additional recurrence of CMO was observed at eighteen weeks following the second Ozurdex. Following a third Ozurdex injection visual acuity deteriorated (from logMAR 0.74 to 0.78) and CMT decreased (from 332 *μ*m to 279 *μ*m). *Conclusion*. Treatment of macular oedema secondary to MacTel with Ozurdex demonstrated promising anatomical outcomes. However, visual outcomes continued to gradually deteriorate.

## 1. Introduction

Macular telangiectasia (MacTel) is rare retinal vascular disorder characterised by irregular dilatations of the capillary network affecting the macula [1]. It can be caused by various retinal vascular inflammatory or occlusive diseases such as diabetic retinopathy, hypertensive retinopathy, or venous occlusion [1]. Additionally, there are other forms which have no known cause; this form of MacTel was first described by Gass and Oyakawa in 1982 and is currently known as idiopathic macular telangiectasia (IMT) [1, 2].

Recent advances in angiographic imaging and optical coherence tomography have led to greater understanding of IMT [3]. Based on these findings, IMT has recently been classified by Yannuzzi et al. into three categories [3]:

- Type 1: aneurysmal telangiectasia;
- Type 2: idiopathic perifoveal telangiectasia;
- Type 3: occlusive telangiectasia.

Type 1 IMT most commonly occurs in males and is unilateral [3]. The age at presentation is frequently between 40 and 70; it has a strong association with systemic hypertension [3]. Features include multiple, venular, and arteriolar aneurysms which are present in both superficial and deep retinal capillary circulations, patchy capillary ischaemia, and lipid deposition [3]. Visual loss predominantly occurs as a result of macular oedema and exudation [4].

Although several treatments have been suggested for the treatment of type 1 IMT, such as laser photocoagulation, intravitreal steroids (e.g., triamcinolone), and antivascular endothelial growth factor (anti-VEGF) agents (e.g., bevacizumab), no treatment has yet been proven to provide consistent visual outcomes [5–8].

Type 2 IMT most commonly presents bilaterally and is characterised in its early stages by retinal transparency in the temporal juxtafoveolar [4]. It presents equally between males and females and is most common in patients who are middle-aged or older [4]. It is also associated with diabetes mellitus and systemic hypertension [4]. Clinical characteristics are blurred vision, metamorphopsia, or a paracentral scotoma [4]. Subretinal neovascularisation is evident in its late stages, often present temporally resulting from retinal capillary remodelling, proliferation, and invasion of the outer retina which has progressively atrophied [4].

Type 3 IMT is a rare form of IMT [4]. It is associated with visual loss in association with systemic or cerebral familial disease [4]. It is characterised by progressive bilateral proliferative capillary obliteration, capillary telangiectasis, and minimal exudation [4].

Dexamethasone intravitreal implant (Ozurdex) has been recently introduced for the treatment of macular oedema following branch retinal vein occlusion or central retinal vein occlusion [9, 10]. It has also been implicated in treatment of other causes of macular oedema including diabetic retinopathy and Irvine-Gass syndrome [11–13].

This case report discusses a patient with macular oedema related to type 1 idiopathic macular telangiectasia which was recalcitrant to treatment with intravitreal injections of bevacizumab and triamcinolone. Subsequently, intravitreal dexamethasone injection (Ozurdex) implant was used with visual and anatomical results presented. This is a novel treatment for macular telangiectasia with macular oedema, with only one published case to the best of our knowledge [14].

## 2. Case Report

A 65-year-old female presented to the Ophthalmology Clinic complaining of deterioration and distortion of vision in her right eye over the last 2 months. She was referred by a Speciality Doctor in Ophthalmology who noted a previous diagnosis of an epiretinal membrane in the right eye and vitreomacular traction. The left eye had stable findings for dry age-related macular degeneration. There was no other ocular history. Systemic history included high cholesterol level, hypertension, fibromyalgia, hypothyroidism, atrial fibrillation, and 8 occurrences of transient ischaemic attacks in the past 9 years. Regular medication included warfarin, simvastatin 80 mg, perindopril 2 mg, bisoprolol 10 mg, amitriptyline 30 mg, levothyroxine 75 mg, and fluoxetine 40 mg. The patient was noted to be allergic to penicillin and septrin. There was no relevant family history of note.

**Figure 1:**
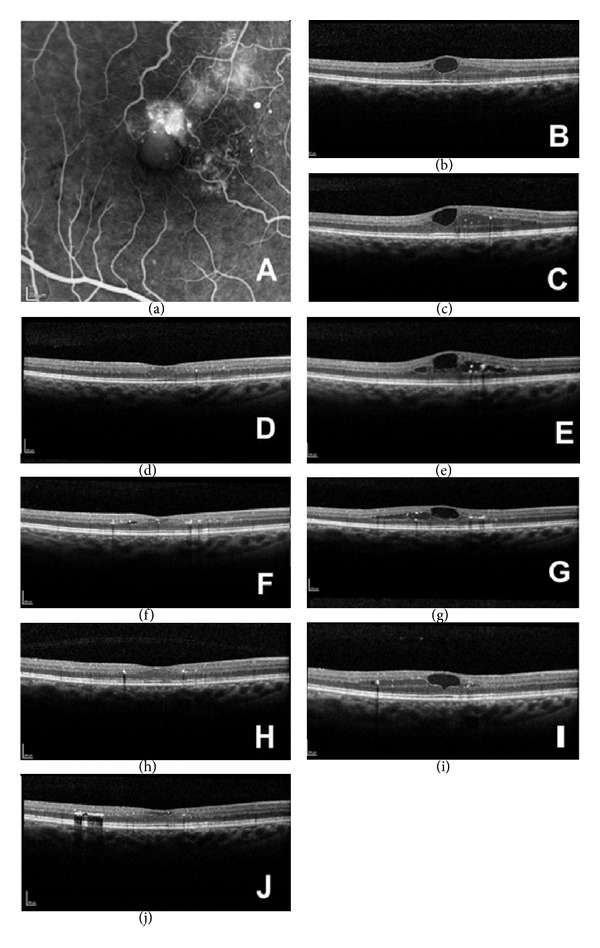
(a) Fundus fluorescein angiography showing perifoveal macular telangiectasia. (b) Ocular coherence tomography (OCT) of the right eye at presentation showing cystoid macular oedema, CMT = 353 *μ*m. (c) OCT of the right eye on clinic visit following a course of three intravitreal bevacizumab injections (one each consecutive month) showing cystoid macular oedema, CMT = 401 *μ*m. (d) OCT of the right eye on clinic visit following intravitreal triamcinolone injection showing no cystoid macular oedema, CMT = 282 *μ*m. (e) OCT of the right eye on second clinic visit following intravitreal triamcinolone injection showing recurrence of cystoid macular oedema, CMT = 397 *μ*m. (f) OCT of the right eye on clinic visit following intravitreal injection of Ozurdex implant showing reduced cystoid macular oedema, CMT = 286 *μ*m. (g) OCT of the right eye on second clinic visit following intravitreal injection of Ozurdex implant showing a recurrence of cystoid macular oedema, CMT = 349 *μ*m. (h) OCT of the right eye on clinic visit following second intravitreal injection of Ozurdex implant showing a reduction in cystoid macular oedema, CMT = 279 *μ*m. (i) OCT of the right eye on clinic visit at four and a half months following second intravitreal injection of Ozurdex implant showing a recurrence of cystoid macular oedema, CMT = 332 *μ*m. (j) OCT of the right eye on clinic visit at two weeks following a third intravitreal injection of Ozurdex implant showing reduced cystoid macular oedema, CMT = 279 *μ*m.

On her first clinic visit, best corrected visual acuity (BCVA) was logMAR 0.30 for the right and 0.00 for left eye. Slit lamp biomicroscopy of the anterior segment was normal. Fundus exam of the right eye revealed cystoid macular oedema which was confirmed on ocular coherence tomography (OCT) (Figure 1(b)). Central macular thickness (CMT) was measured to be 353 *μ*m (Table 1). Fundus fluorescein angiography (FFA) was performed showing evidence of macular teleangiectasia type 1 in the right eye (Figure 1(a)).

Initial treatment included a course of 3 intravitreal bevacizumab (IVB) (Avastin) injections (one each consecutive month). Six weeks posttreatment with IVB, BCVA of her right eye was logMAR 0.18. OCT of the right eye showed increase in CMO, CMT recorded as 401 *μ*m (Figure 1(c)) (Table 1). As the response to anti-VEGF treatment was poor, the decision was made to treat with a single intravitreal triamcinolone injection (IVTA) [5].

Two weeks posttreatment with IVTA, there was deterioration in vision of the right eye, BCVA being logMAR 0.48. However, there was a reduction in CMT (recorded as 282 *μ*m) (Figure 1(d)). No further treatment was decided at the time. At 6 weeks posttreatment with IVTA, there was no improvement in BCVA and there was a recurrence in CMO, CMT recorded as 397 *μ*m (Figure 1(e)) (Table 1). The patient was then recommended to have treatment with a single intravitreal dexamethasone implant (Ozurdex).

A standard protocol was followed for the intravitreal injection of Ozurdex 700 mcg, including topical anaesthesia, installation of povidone iodine 5%, sterile drape, and lid speculum. No peri- or postoperative complications were observed. Six weeks posttreatment with the Ozurdex implant, BCVA of the right eye deteriorated to logMAR 0.59. However, OCT scan showed decreased CMT of 286 *μ*m (Figure 1(f)). No further treatment was decided at the time.

Fourteen weeks posttreatment with Ozurdex, a further decrease in visual acuity was found; logMAR was 0.7. OCT demonstrated new occurrence of CMO, CMT recorded as 349 *μ*m (Figure 1(g)) (Table 1). Patient was listed for a second intravitreal injection of Ozurdex.

Six weeks posttreatment with the second Ozurdex, BCVA of the right eye improved to logMAR 0.64. OCT showed a decrease of CMO, CMT recorded as 279 *μ*m (Figure 1(h)) (Table 1). Eighteen weeks posttreatment with the second Ozurdex, BCVA deteriorated to logMAR 0.74, BCVA deteriorated to logMAR 0.74. There was a recurrence of CMO, CMT recorded as 332 *μ*m (Figure 1(i)). Patient was listed for a third Ozurdex injection. Two weeks posttreatment with the third Ozurdex injection, the patients' visual acuity was recorded as 0.78; CMT decreased to 279 *μ*m (Figure 1(j)).

During the whole period of follow-up, no ocular adverse events were established. No cataract progression was noted and intraocular pressure remained within normal limits.

## 3. Discussion

The treatment of type 1 idiopathic macular telangiectasia remains challenging as no established treatment currently exits [4].

Recently, studies examining the use of intravitreal injections of bevacizumab as a treatment for this condition have found it to be inconsistent in its outcomes [7, 15–18]. Only a few cases have been reported on the use intravitreal injections of triamcinolone acetonide with promising results [5]. However, high complication rates including elevated intraocular pressure and cataract have limited its use [19, 20].

The benefits of using corticosteroids for treating macular oedema due to type 1 IMT arises due to their anti-inflammatory action, their stabilisation of the blood-retinal barrier, and their prohibition of vascular endothelial growth factor action [4]. The Ozurdex implant provides progressive release of dexamethasone, leading to enhanced anti-inflammatory action when compared with intravitreal injections of triamcinolone. With regard to safety, Ozurdex has demonstrated a reasonable safety profile. When compared with sham injections, in patients with retinal vein occlusion, adverse events occurring significantly more in Ozurdex patients were ocular hypertension (4%), eye pain (7.4%), and anterior chamber cells (1.2%) [9].

Our case report demonstrates that treating type 1 IMT with Ozurdex leads to good anatomical outcomes, compared with intravitreal triamcinolone recurrences of CMO were reduced in magnitude (as demonstrated by CMT) and the period without recurrences was prolonged following treatment with Ozurdex. Visual acuity continued to deteriorate following treatment with the Ozurdex; however, the rate of deterioration was of less magnitude when compared to intravitreal triamcinolone. In addition, a reduction in the number of intravitreal injections was achieved with Ozurdex compared to intravitreal bevacizumab, decreasing the risk for adverse events from intravitreal injections such as endophthalmitis.

This is the second case report published demonstrating promise from the treatment of type 1 IMT with Ozurdex, although for a case with differing characteristics [14]. A prospective study evaluating Ozurdex for type 1 IMT will provide insight into whether it should be regarded as a more established method of treatment.

## Figures and Tables

**Table 1:** Table demonstrating visual acuity (measured using logMAR) and central macular thickness (CMT) measured using ocular coherence tomography in right eye of patient with macular telangiectasia type 1. Data is demonstrated at baseline, 2–6 weeks following each treatment, and after a recurrence of cystoid macular oedema (CMO).

Treatment	logMAR following treatment	CMT following treatment (*μ*m)	Time for recurrence of CMO after treatment	logMAR at recurrence of CMO	CMT at recurrence of CMO (*μ*m)
At baseline	0.3	353	N/A	N/A	N/A
Intravitreal bevacizumab	0.18	401	N/A	N/A	N/A
Intravitreal triamcinolone	0.48	282	8 weeks	0.48	397
Ozurdex	0.59	286	14 weeks	0.70	349
Ozurdex	0.64	279	18 weeks	0.74	332
Ozurdex	0.78	279	N/A	N/A	N/A
